# Theory of Non-Equilibrium Heat Transport in Anharmonic Multiprobe Systems at High Temperatures

**DOI:** 10.3390/e23121630

**Published:** 2021-12-03

**Authors:** Keivan Esfarjani

**Affiliations:** 1 Department of Mechanical and Aerospace Engineering, University of Virginia, Charlottesville, VA 22904, USA; k1@virginia.edu; 2 Department of Materials Science and Engineering, University of Virginia, Charlottesville, VA 22904, USA; 3 Department of Physics, University of Virginia, Charlottesville, VA 22904, USA

**Keywords:** nanoscale thermal transport, anharmonicity, phonons, heat current, transmission, thermal conductance

## Abstract

We consider the problem of heat transport by vibrational modes between Langevin thermostats connected by a central device. The latter is anharmonic and can be subject to large temperature difference and thus be out of equilibrium. We develop a classical formalism based on the equation of motion method, the fluctuation–dissipation theorem and the Novikov theorem to describe heat flow in a multi-terminal geometry. We show that it is imperative to include a quartic term in the potential energy to insure stability and to properly describe thermal expansion. The latter also contributes to leading order in the thermal resistance, while the usually adopted cubic term appears in the second order. This formalism paves the way for accurate modeling of thermal transport across interfaces in highly non-equilibrium situations beyond perturbation theory.

## 1. Introduction

Transport theories of non-interacting quantum systems based on the Keldysh formalism, which treats non-equilibrium flow of charge or heat carriers in a one-dimensional (1D) geometry have been developed in the past. To the best of our knowledge, the first such development was performed in 1971 by Caroli et al. [1] where a Green’s function formalism was used to describe dynamics of electrons in a 1D crystal. Following the seminal work of Caroli et al., many other groups worked on similar formalisms and proved a formula for the transmission through the system, now widely used for both non-interacting electrons and phonons. The equilibrium version of it, namely $T=\mathrm{Tr}[G\Gamma_{L}G^{†}\Gamma_{R}]$, where *G* and Γ are, respectively, the retarded Green’s function and the escape rates to the leads, was established by Meir and Wingreen [2] and in a similar form by Pastawski [3] in 1991. This formula holds for a non-interacting (or harmonic, in the case of phonons) system *near* equilibrium, meaning the chemical potential or temperature gradients are to be infinitesimally small. These assumptions might not always be realistic, especially in small (mesoscopic) systems subject to temperature differences over fractions of a micrometer, and a formulation for non-equilibrium situations and interacting systems is preferable for the sake of testing the domain of validity of the equilibrium formulas and more accurate description in the case of large interactions and large driving fields.

**Figure 1:**
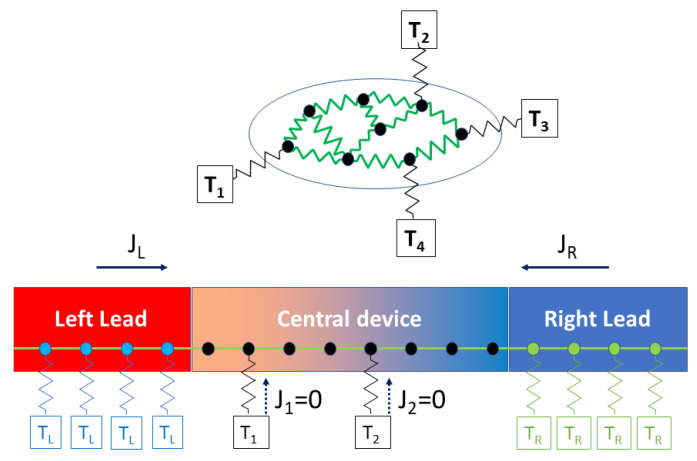
(**Top**) a general “molecular” multiprobe geometry where the device defined by atoms within the ellipse are connected to 4 reservoirs imposing a temperature or measuring a current. (**Bottom**) the 2-probe geometry. Atoms in each lead are connected to a harmonic thermostat at fixed temperature $T_{L}$ and $T_{R}$. In the Buttiker probe, also called the self-consistent reservoirs geometry, to measure the “local” temperature, each layer of the central device may also be weakly connected to a fictitious thermostat at a temperature to be (self-consistently) determined so that the net heat flow from that probe to the device is zero.

The general and practical problem of interest is the description of the heat transport across interfaces between different materials; however, to keep it more general, we adopt a multi-probe geometry where the system in which scatterings occur is connected to multiple energy reservoirs, which impose their temperature, and cause the flow of heat carriers (see Figure 1). This model is used for mesoscopic systems where the carrier mean free path could be on the order of the system length, implying Ohm’s law of addition of resistances in series does not necessarily hold, and coherence can play an important role. The geometry of the reservoirs is fundamentally one-dimensional (1D), and if there is translational symmetry perpendicular to the current flow, one can use Bloch’s theorem to decouple the 3D system into many non-interacting 1D systems, each labeled by a quantum number, which is the transverse momentum. So in what follows, we assume such decoupling has been performed and we will be dealing with strictly 1D semi-infinite harmonic leads, although the central device is arbitrary in shape and structure and maybe connected to multiple 1D probes, assumed to be Langevin thermostats and assumed to be isothermal.

For simplicity, we will use a *classical* description. A generalization to the quantum case will be inferred at the end. So in our classical treatment, the frequency ω is just frequency of vibrational modes and not the energy of phonons. This classical formalism avoids fancier mathematics involving commutation relations, and concepts such as time-ordered or contour-ordered Green’s functions. It will only involve “retarded” or causal Green’s functions, which help us solve a differential equation in the frequency domain. In the high-temperature limit both theories lead in principle to the same results. To model interfacial thermal resistance, typical considered geometries will be identical to a non-equilibrium molecular dynamics (NEMD) setup where the two ends of the system are attached to two thermostats at different temperatures, and one is interested in measuring the established heat flow as a result of the temperature difference (see Figure 1).

The non-equilibrium anharmonic phonon problem has been addressed in the past by Mingo [4] and separately by Wang [5] in 2006. They used the many-body perturbation approach of non-equilibrium quantum systems based on the Keldysh formalism, (also called the Non-Equilbrium Green’s Function or NEGF method) and derived a lowest-order approximation for the transmission function. Dai and Tian [6] recently implemented this rigorous formulation to calculate the effect of cubic anharmonicity on the phonon transmission function through an ideal interface, applied to Si/Ge and Al/Al with two different masses. A similar model, which explicitly incorporates transverse momentum dependence, was also developed recently by Guo et al. [7], based on previous work by Luisier [8]. Polanco has a recent review of these methods based on NEGF [9]. These NEGF-based models, although fully quantum mechanical, do not include anharmonicity beyond cubic order nor any thermal expansion effects. Other calculations of the transmission in the non-equilibrium regime [10] based on the Green’s function method, have been based on the self-consistent reservoirs (also called Buttiker probe method), which was first proposed by Bolsterli et al. in 1970 [11]. In this method, as shown in Figure 1, every layer is connected, with a *very weak* coupling, to a fictitious probe at a given temperature with which it becomes into equilibrium. The probe temperature, which is also the assigned temperature of that layer, is obtained from the constraint that the net heat current from the system to the added fictitious probe should be zero. Note that the system could be out of equilibrium, so that a temperature is not really well-defined at a given layer. This shortcoming is also present in NEMD simulations where the assigned “temperature” of a layer is just the average kinetic energy of atoms in that layer, although there is no evidence of local thermal equilibrium. So even though non-equilibrium effects are included through this Buttiker probe method, it does not include anharmonicity. We should mention at this point that there is numerical evidence of absence of equipartition in the vibrational modes near the interface [12,13], implying that a definition of local temperature is not really justified near an interface. In another work, based on the MD simulation, which fully includes anharmonicity, Saaskilahti et al. [14] extended the harmonic formulation of the transmission function based on the actual MD trajectories by Chalopin et al. [15,16], to include anharmonic corrections. In the actual calculation, arguing that the anharmonic part of the current is usually small, they only used its harmonic formula but with velocities and positions coming from the full anharmonic atomic trajectories, in order to deduce the interfacial thermal conductance. The advantage of this approach over NEMD is that the heat current and thermal conductance can be decomposed in the frequency domain. Approaches based on MD trajectories, while including full anharmonicity, suffer from noise and would require a large number of simulations in order to perform proper ensemble averaging, whereas many-body approaches might be inaccurate if a perturbative expansion in powers of anharmonicity is used, but otherwise do not suffer from noise and treat ensemble averaging analytically. In this work, we try to overcome these limitations by adopting a non-perturbative many-body approach by fully including in the current the effect of anharmonic terms introduced in the Hamiltonian, without using Buttiker probes. Furthermore, using a classical method to derive an expression for the heat current, we argue that to leading order, it is necessary to include quartic terms in the Hamiltonian, in order to properly describe both the thermal expansion and the dominant temperature dependence effect in the heat current.

## 2. Dynamics

We start by defining our model and the assumptions. A multiprobe geometry is assumed as shown in Figure 1 in which a central region, also called the “device” is connected to many semi-infinite one-dimensional (1D) leads, playing the role of thermostats imposing a temperature at the boundaries of the system. We will not be concerned with temperature drops in the thermostats, which are assumed to be harmonic and follow Langevin dynamics. If needed, parts of the leads can be incorporated in the device region to illustrate the temperature drops near the interface. The device (*D*) Hamiltonian is a fourth-degree polynomial in powers of atomic displacements, and it is harmonically coupled to the lead α is represented by Equation (2):

(1) $$\begin{array}{cc} H_{D} & =\sum_{i\in D}\frac{p_{i}^{2}}{2m_{i}}+\sum_{ij\in D}\;\frac{1}{2!}\phi_{ij}\;u_{i}\;u_{j}+\sum_{ijk\in D}\;\frac{1}{3!}\psi_{ijk}\;u_{i}\;u_{j}\;u_{k}+\ldots \end{array}$$

(2) $$\begin{array}{cc} H_{\alpha ,D} & =\sum_{i\in D}\sum_{l\in \alpha }\;W_{\alpha ,il}\;u_{i}\;u_{l} \end{array}$$

The dynamical variable $u_{i}\left(t\right)$ refers to the displacement of atom *i* about the zero-temperature equilibrium position (the force on atom is zero for u=0). The leads labeled by α are semi-infinite harmonic chains. After the standard change of variables to $x_{i}\left(t\right)=\sqrt{m_{i}}u_{i}\left(t\right)$, and to

$$\Phi_{ij}=\frac{\phi_{ij}}{\sqrt{m_{i}m_{j}}}=\frac{1}{\sqrt{m_{i}m_{j}}}\frac{\partial^{2}H_{D}}{\partial u_{i}\partial u_{j}}\left(u=0\right)$$

$V_{\alpha ,il}=W_{\alpha ,il}/\sqrt{m_{i}m_{l\alpha }}$ and $\Phi_{\alpha ,ll^{\prime }}=\phi_{\alpha ,ll^{\prime }}/m_{l\alpha }$ we arrive at the following equation of motion for atoms in the central region:

(3) $$\frac{d^{2}x}{dt^{2}}=-\Phi \;x-\sum_{\alpha }V_{\alpha }\;x_{\alpha }+a$$

Note we have used capitalized Greek letters (Φ,Ψ,…) for mass-rescaled force constants, and lower-case Greek letters (ϕ,ψ,…) for the bare potential energy derivatives. The letter α refers to the leads, and the dynamical variable $x=(x_{1},\ldots ,x_{N})$ can be thought of as an array containing the displacements of all the atoms in the central region (also called device), Φ as the force constant matrix between such atoms, and $V_{\alpha }$ as the force constant matrix connecting atoms of the lead α to atoms in the device. Finally, $a=-1/2\Psi x^{2}+\ldots$ is the anharmonic part of the force, which, for now, we keep as *a* for brevity. The dynamics of atoms in the lead is of *Langevin* type [17], where a set of identical coupled harmonic oscillators are subject to damping $\gamma_{\alpha }$ and noise $\zeta_{\alpha }$. The equation of motion for atoms with displacements $x_{\alpha }$ is as follows:

(4) $$\frac{d^{2}x_{\alpha }}{dt^{2}}=-\Phi_{\alpha }x_{\alpha }-V_{\alpha }^{T}x-\gamma_{\alpha }\frac{dx_{\alpha }}{dt}+\zeta_{\alpha }$$

where the superscript *T* stands for transpose. Here again all atomic degrees of freedom are stored in the array $x_{\alpha }=\left(x_{1\alpha },x_{2\alpha },\ldots \right)$. The force constants $Phi_{\alpha }$ can be thought of as effective FCs at the temperature of interest, so that we do not need to introduce anharmonicity in the leads, which merely play the role of absorbing phonons from the device and reinjecting thermalized phonons into the device. We will proceed by eliminating the lead variables $x_{\alpha }$ in Equation (3) using the Green’s function method. To this end, we start by taking the Fourier transform of the above two equations according to:

(5) $$X\left(\omega \right)=\int dt\;x\left(t\right)\;e^{i\omega t};\;\;x\left(t\right)=\int \;\frac{d\omega }{2\pi }\;X\left(\omega \right)\;e^{-i\omega t}$$

In the frequency domain, the equations of motion (3) and (4) become:

(6) $$\begin{array}{cc} -\omega^{2}X & =-\Phi X-\Sigma_{\alpha }V_{\alpha }\;X_{\alpha }+A \end{array}$$

(7) $$-\omega^{2}X_{\alpha }=-\Phi_{\alpha }X_{\alpha }-V_{\alpha }^{T}X+i\omega \gamma_{\alpha }X_{\alpha }+\zeta_{\alpha }\left(\omega \right)$$

Note that the frequency-domain variables are represented with capitalized letters, and the transient part of the solution will be omitted as we are interested in the steady-state solution. Now that the differential equations are transformed to algebraic ones, one can easily proceed to eliminate the lead degrees of freedom in the main equation of motion by using Green’s functions. Let $g_{\alpha }\left(\omega \right)$ be the retarded (causal) Green’s function associated with the lead α. The positivity of the damping factor $\gamma_{\alpha }$ insures causality. The solution to Equation (7) after the transients have decayed to zero, can be written as:

(8) $$X_{\alpha }=g_{\alpha }\left(\omega \right)\;\left(\zeta_{\alpha }-V_{\alpha }^{T}X\right)$$

where

(9) $$g_{\alpha }^{-1}=\left[-\omega^{2}-i\omega \gamma_{\alpha }+\Phi_{\alpha }\right]$$

In Equation (6) we need $-V_{\alpha }X_{\alpha }$, which we can obtain from Equation (8):

(10) $$-V_{\alpha }X_{\alpha }=\eta_{\alpha }+\sigma_{\alpha }X,$$

Here $\eta_{\alpha }\left(\omega \right)=-V_{\alpha }g_{\alpha }\left(\omega \right)\zeta_{\alpha }\left(\omega \right)$, and $\sigma_{\alpha }=V_{\alpha }\;g_{\alpha }V_{\alpha }^{T}$. Likewise, defining the retarded Green’s function of the central region as $G^{-1}=\left[-\omega^{2}+\Phi -\sum_{\alpha }\sigma_{\alpha }\left(\omega \right)\right]$, we can write the solution to the central region as:

(11) $$X\left(\omega \right)=G\left(\omega \right)\;\left[\sum_{\alpha }\eta_{\alpha }\left(\omega \right)\;+A\left(\omega \right)\right]$$

The function $\sigma_{\alpha }\left(\omega \right)=V_{\alpha }\;g_{\alpha }\left(\omega \right)V_{\alpha }^{T}$ is traditionally called the self-energy of lead α, and shows the effect of this lead on the spectrum of the device, which is given by the poles of *G*. Its real part provides a correction to the eigenvalue spectrum $\omega^{2}$, and its imaginary part, divided by 2ω, gives the inverse lifetime of an excitation of the central region caused by interactions with the lead. It is the rate at which the excitation leaks into the lead. Omitting the transient contribution of the initial conditions, this is the solution to the equations of motion, which depend on the stochastic functions $\eta_{\alpha }=-V_{\alpha }g_{\alpha }\zeta_{\alpha }$.

## 3. Physical Observables

The equations of motion we derived are deterministic for every realization of the random forces. To simulate the real thermodynamical behavior of the baths, so that a temperature can be assigned to them, one needs to perform an “ensemble” average, denoted by 〈⋯〉 over all realizations of the forces subject to the constraint imposed by the fluctuation–dissipation (FD) theorem [17]:

$$\langle \zeta_{i,\alpha }\left(t\right)\rangle =0$$

(12) $$\langle \zeta_{i,\alpha }\left(t\right)\zeta_{j,\alpha^{\prime }}\left(t^{\prime }\right)\rangle =2\gamma_{\alpha }k_{B}T_{\alpha }\;\delta \left(t-t^{\prime }\right)\delta_{\alpha ,\alpha^{\prime }}\delta_{ij}$$

The noise is white and different sites i,j of leads $\alpha ,\alpha^{\prime }$ are uncorrelated with each other. Physical observables are then obtained after an ensemble average is performed over forces. This is where irreversibility is introduced in this deterministic formalism, as a result of which, entropy is generated in the device. The latter can be understood as the log of the distribution function of the device as the random forces are varied within the constraints imposed by the FD theorem.

### 3.1. Entropy Generation Rate

An important quantity of interest is the entropy generation rate in the device, which can be expressed as: $\dot{S}=-\sum_{\alpha }\langle j_{\alpha }\rangle /T_{\alpha }$. This quantity results from the interaction between the device degrees of freedom and the random noise due to Langevin thermostats. It involves the thermostats temperature and the incoming currents which will be discussed next.

### 3.2. Heat Current

The main quantity of interest is the heat current. The heat from lead α can be defined as the net rate at which energy is flowing into the device from that lead. It is the work performed from lead α on the device’s degrees of freedom per unit time, which is the product of the velocity degrees of freedom of the device times the force from lead acting on them: $j_{\alpha }\left(t\right)=\mathrm{Tr}\left[\dot{x}\;{\left(-V_{\alpha }x_{\alpha }\right)}^{T}\right]$, where the trace is taken over the device degrees of freedom. Substituting the expressions for *x* and $x_{\alpha }$ from Equations (10) and (11) we can obtain the final expression of the current in the frequency domain. Since the current depends on the stochastic functions η, we will take its ensemble average to find the response of the system to an applied temperature difference. We will start by taking the Fourier transform of $j_{\alpha }\left(t\right)$ and call it $J_{\alpha }\left(\Omega \right)$. Note due to time integration, we must have $\langle J_{\alpha }\left(\Omega =0\right)\rangle =\tau \langle j_{\alpha }\rangle$, where τ→∞ is the time integration window. Finally the static DC current is given by:

(13) $$\langle j_{\alpha }\rangle =\int \frac{d\omega }{2\pi \tau }\;\omega \;\mathrm{Tr}\;\left[ℑ\langle X\left(\omega \right)\eta_{\alpha }^{†}\left(\omega \right)\rangle -\langle X\left(\omega \right)X^{†}\left(\omega \right)\rangle \;\Gamma_{\alpha }\left(\omega \right)/2\right]$$

where we used the notation $\Gamma_{\alpha }=-i\left(\sigma_{\alpha }-\sigma_{\alpha }^{†}\right)=2ℑ\left(\sigma_{\alpha }\right)$ for twice the imaginary part of the lead α self-energy. The DC response is found by taking the Ω→0 limit. Note that because we are interested in the DC response, only diagonal terms of correlations (Ω=0) are needed here. Thus the calculation of the heat current is reduced to the calculation of the two correlation functions and the so-called lead self-energy $\sigma_{\alpha }\left(\omega \right)$, followed by a frequency integration. Note that this current from lead α is the sum of two terms: the first one, proportional to $Z_{\alpha }=\langle X\left(\omega \right)\;\eta_{\alpha }^{†}\left(\omega \right)\rangle$, is the work per unit time of the stochastic forces on the device, while the second one $\langle X\left(\omega \right)\;X^{†}\left(\omega \right)\rangle \Gamma_{\alpha }^{†}$, proportional to a displacement autocorrelation, is the work of the lead dampers trying to reabsorb some of the excess energy injected from the device in order to re-establish thermal equilibrium in the lead.

The average denoted by 〈〉 is an average over the stochastic forces in the thermostats that have white noise characteristics. When it is performed over the device degrees of freedom, the leads being at different temperatures, it becomes a *non-equilibrium* average and can only be calculated using the equation of motion (11) and the statistical properties of the Langevin thermostats, i.e., the fluctuation–dissipation theorem. For anharmonic interactions involving higher powers of displacements in *A*, the calculation of current will lead to a hierarchy of equations, each containing higher powers of displacements, and has so far been computed using different approximations [4,5,18].

## 4. Constraints

Before proceeding to the calculation of the correlation functions, we recall two constraints that the heat current needs to satisfy. The first is the *detailed balance* relation, which states that if all leads are at the same temperature *T*, the net current $\langle j_{\alpha }\rangle$ should be identically zero for all leads α. The second constraint is that of *current conservation*, which in steady state Ω→0, and under no additional heat generation in the device, reduces to $\Sigma_{\alpha }\langle j_{\alpha }\rangle =0$. This is also known as the *Kirchhoff’s law* in the context of electrical circuits. Note that AC components of the current need not satisfy this constraint as they reflect the information on transient currents during the relaxation process, and depend on the heat capacity of the system. Any physically correct description of transport should exactly satisfy these two constraints.

## 5. Thermal Expansion

As the temperature of a system is raised, there can be thermal expansion due anharmonicity. The equilibrium position of the atoms is shifted, and this will also cause a change in the force constants as bond lengths have changed. To take these effects into account, while simplifying the notations, we will slightly modify the formalism as follows: the displacement variable *X* is changed to Y(ω)=X(ω)−〈X〉 or y(t)=x(t)−〈x〉 which has zero average by construction. The resulting nonlinear equations satisfied by 〈X〉 are derived by taking the average of the equation of motion (11) or equivalently setting the average force on each atom to zero (see Appendix B for more details).

(14) 〈∂V/∂x〉=Φ〈x〉+Ψ〈xx〉/2+…=0

The resulting equations will depend on correlations such as $\langle Y_{i}Y_{j}\rangle$, $\langle Y_{i}Y_{j}Y_{k}\rangle$ and higher powers. Accordingly, the potential energy derivatives will be evaluated at the zero of *Y*, and will be denoted with a bar sign on top of them ($\Phi \to \bar{\Phi }$ etc…). While the variable *X* satisfies the equation of motion:

$$-\omega^{2}X=-\partial V/\partial X-\sum_{\alpha }V_{\alpha }X_{\alpha }\;\;,$$

the new variable *Y* satisfies:

$$-\omega^{2}Y=-\partial V/\partial X+\langle \partial V/\partial X\rangle -\sum_{\alpha }V_{\alpha }X_{\alpha }$$

Next we will linearize the forces with respect to *y*:

$$\begin{array}{c} -\frac{\partial V}{\partial y}=-{\left(\frac{\partial V}{\partial y}\right)}_{y=0}-{\left(\frac{\partial^{2}V}{\partial y^{2}}\right)}_{y=0}\;y+a\left(y\right)=-\bar{\Phi }y+a\left(y\right) \end{array}$$

where we have set ${\left(\frac{\partial V}{\partial y}\right)}_{y=0}=0$ to define the thermal expansion $\langle x\rangle =x_{0}$ (see Appendix B). As we will show, the effect of temperature will be to renormalize the FCs, not only through thermal expansion but also due the thermal fluctuations as we will show using the non-equilibrium mean-field approximation (NEMF) also detailed in Section 6. Next, we define a renormalized Green’s function using renormalized force constants $\bar{\Phi }={\left(\frac{\partial^{2}V}{\partial y^{2}}\right)}_{y=0}$ as

(15) $$G^{-1}=\left[-\omega^{2}+\bar{\Phi }-\sum_{\alpha }\sigma_{\alpha }\right]$$

With this GF, the displacements *Y* satisfy

(16) $$Y=G(\sum_{\alpha }\eta_{\alpha }+A)$$

Note one can add any constant λ to the force constant $\bar{\Phi }$ in the above GF, provided λY is also added to the anharmonic force *A*. We will make use of this freedom in the next section to further simplify the formalism.

## 6. Force Constant Renormalization

Given the form of the above equations, we can add $-Y\langle \frac{\partial A}{\partial Y}\rangle$ to *A* and add $-\langle \frac{\partial A}{\partial Y}\rangle$ to 1/G so that now the renormalized GF becomes:

(17) $$G^{-1}=G^{-1}-\langle \frac{\partial A}{\partial Y}\rangle =\left[-\omega^{2}+\bar{\Phi }-\langle \frac{\partial A}{\partial Y}\rangle +\sum_{\alpha }\sigma_{\alpha }\right]$$

while the renormalized anharmonic force now becomes $A=A-Y\langle \frac{\partial A}{\partial Y}\rangle$ in the right hand side of Equation (16). This renormalization of harmonic force constants captures a major part of anharmonicity (because 〈∂A/∂Y〉=0), and is in spirit very similar to the lowest-order self-consistent phonon theory, which in the past has been applied to equilibrium systems. The advantage of this renormalization is that, as we will see, the lowest anharmonic correction in $Z_{\alpha }$ disappears by construction since 〈∂A/∂Y〉=0, leading to the smallest variance and higher moments of ∂A/∂Y.

We will refer to this choice of the reference GF as the *Non-equilibrium mean-field* approximation (NEMF). With this choice, the equation of motion for *Y* becomes:

(18) $$\begin{array}{c} Y=G(\sum_{\alpha }\eta_{\alpha }+A) \end{array}$$

**Figure 2:**
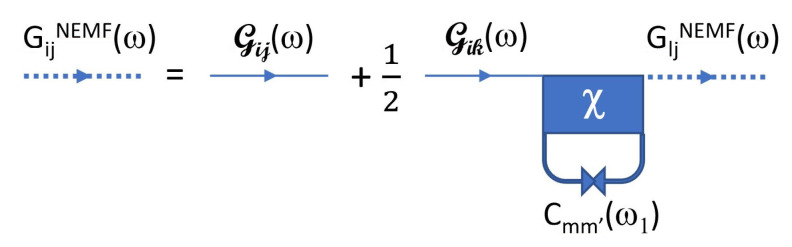
Feynman diagram associated with G represented by thick dashed lines. The phonon Green’s function G is shown with thin solid lines, and the quartic vertex χ with the solid square. The thick line with opposite arrows represents the displacement autocorrelation $C(\omega_{1})$. Internal frequency $\omega_{1}$ is integrated over.

The Feynman diagram associated with the new Green’s function G is shown in Figure 2.

## 7. Displacement-Noise Correlations

One can see from Equation (13) that the calculation of the heat current requires the calculation of the displacement-noise correlation $Z_{\alpha }$ and displacement autocorrelations *C*. We proceed to the calculation of these quantities first within the harmonic approximation, and then in the presence of anharmonic forces of the form $a=-\psi \;y^{2}/2-\chi \;y^{3}/6$.

Let us start with the noise autocorrelation, which will appear in the calculation of displacement-noise correlation $Z_{\alpha }$. In the frequency domain, using the fluctuation–dissipation theorem Equation (12), one can derive (see Appendix C.2):

(19) $$\begin{array}{c} \begin{array}{c} \langle \eta_{\alpha }\left(\omega \right)\eta_{\alpha }^{†}\left(\omega \right)\rangle =\Gamma_{\alpha }\left(\omega \right)\frac{k_{B}T_{\alpha }}{\omega }\tau =\Gamma_{\alpha }\left(\omega \right)f_{\alpha }\tau \end{array} \end{array}$$

where, for brevity, we have replaced the “occupation factor” $k_{B}T_{\alpha }/\omega$ by $f_{\alpha }$, and τ represents the integration time which goes to infinity and cancels the τ in the expression for the current $j_{\alpha }=J_{\alpha }/\tau$. With a Factor of *ℏ* in the denominator of $f_{\alpha }$, the latter would be a real Bose-Einstein occupation factor taken to the classical limit.

In the case of a white noise, we show in Appendix C.2 that the result will not depend on the thermostat damping parameter γ, and thus we adopt this type of noise for the thermostats.

Next, we need to calculate displacement-noise correlations: $Z_{\alpha }=\langle Y\eta_{\alpha }^{†}\rangle$. Since −iωY is a velocity, $\omega ℑ(Z_{\alpha })$ is the power exerted by the random force η on the device and therefore can be interpreted as the *heat injected per unit time and unit frequency (mode) from lead α into the device*.

In the harmonic case (A=A=0;G=G=G), using the equation motion (18), this expression is simplified to:

(20) $$Z_{\alpha }^{H}\left(\omega \right)=G\langle \eta_{\alpha }\;\eta_{\alpha }^{†}\rangle =G\Gamma_{\alpha }f_{\alpha }\tau$$

For non-zero anharmonicity, the three GFs are different, and adopting G as the reference GF, we have the NEMF approximation to the displacement-noise correlation function as:

(21) $$Z_{\alpha }^{NEMF}\left(\omega \right)=G\langle \eta_{\alpha }\;\eta_{\alpha }^{†}\rangle =G\Gamma_{\alpha }f_{\alpha }\tau$$

To go one step further and include the effect of anharmonicity in $Z_{\alpha }$, we will use the Novikov–Furutsu–Donsker identity [19] (for a proof also see Appendix D), which states that for any functional of the white noise f[η], we have

(22) $$\begin{array}{c} \langle f\left[\eta \right]\;\eta_{\alpha }^{†}\left(\omega \right)\rangle =\langle \frac{\delta f[\eta ]}{\delta \eta_{\alpha }}\rangle \langle \eta_{\alpha }\;\eta_{\alpha }^{†}\rangle \end{array}$$

It has the advantage of lowering the powers of η in *f*. Using this theorem, we have: $\langle Y\eta_{\alpha }^{†}\rangle =\langle \frac{\partial Y}{\partial \eta_{\alpha }}\rangle \langle \eta_{\alpha }\;\eta_{\alpha }^{†}\rangle$.

From the equation of motion Equation (18) and the chain rule, we find

$$\begin{array}{c} \frac{\partial Y}{\partial \eta_{\alpha }}={\left(1-G\frac{\partial A}{\partial Y}\right)}^{-1}G=G+G\frac{\partial A}{\partial Y}G+G\frac{\partial A}{\partial Y}G\frac{\partial A}{\partial Y}G+\ldots \end{array}$$

so that finally,

(23) $$\begin{array}{ccc} Z_{\alpha } & = & \langle Y\eta_{\alpha }^{†}\rangle =\langle \frac{\partial Y}{\partial \eta_{\alpha }}\rangle \langle \eta_{\alpha }\;\eta_{\alpha }^{†}\rangle =\langle {\left(1-G\frac{\partial A}{\partial Y}\right)}^{-1}\rangle \;Z_{\alpha }^{NEMF} \end{array}$$

(24) $$\begin{array}{ccc} & = & \left(1+G\langle \frac{\partial A}{\partial Y}G\frac{\partial A}{\partial Y}\rangle +G\langle \frac{\partial A}{\partial Y}G\frac{\partial A}{\partial Y}G\frac{\partial A}{\partial Y}\rangle +\ldots \right)\;Z_{\alpha }^{NEMF} \end{array}$$

Note that, by construction, the first term involving 〈∂A/∂Y〉 is identically zero. This justifies the choice of G rather than G as the reference Green’s function.

In the language of many-body theory, this is the expanded form of Dyson’s equation involving thermal average of powers of the anharmonic force derivatives. This exact result cannot be calculated, because the average of the inverse of powers of *Y* cannot be calculated exactly, and instead one may add the power series term by term provided the sum is convergent.

This is one of the main results of this paper, providing a more accurate and likely convergent expression for the displacement-noise correlations, and leading to a simple calculation of heat currents if cubic terms are to be neglected (using the NEMF reference Green’s function G).

The next-order correction consists in adding the effect of anharmonicity to second order, i.e., writing the displacement-noise correlation function as:

(25) $$Z_{\alpha }\approx \left(1+G\langle \frac{\partial A}{\partial Y}G\frac{\partial A}{\partial Y}\rangle \right)\;Z_{\alpha }^{NEMF}$$

with $A=A-Y\langle \frac{\partial A}{\partial Y}\rangle =-\frac{1}{2}\bar{\Psi }YY-\frac{1}{6}\chi \left(YYY-3Y\langle YY\rangle \right)$ and $\frac{\partial A}{\partial Y}=\frac{\partial A}{\partial Y}-\langle \frac{\partial A}{\partial Y}\rangle =-\bar{\Psi }Y-\frac{1}{2}\chi \left(YY-\langle YY\rangle \right)$. We can note that the major part of the quartic anharmonicity has been removed, and the expansion is in powers of ∂A/∂Y which is centered at zero and has therefore the smallest higher moments. When raised to the second power in the above formula for $Z_{\alpha }$, cubic and quartic terms become decoupled if we neglect averages of terms of odd power in *Y* which are expected to be small if non-zero. At low-temperatures or weak anharmonicity, the dominant contribution to the second-order terms can be written as:

(26) $$Z_{\alpha }\approx \left(1+\langle G\bar{\Psi }YG\bar{\Psi }Y\rangle \right)\;Z_{\alpha }^{NEMF}$$

An explicit form of this equation is provided in the following section and in Appendix C.3 Equation (A17).

## 8. Displacement Autocorrelations

Finally, the last correlation function needed is $C\left(\omega \right)=\langle YY^{†}\rangle$. Note the autocorrelation matrix *C* is Hermitian. The function ωC(ω) can be interpreted as the (non-equilibrium) number of excitations of frequency ω present in the device due to its contact with the leads. If all lead temperatures are equal, we recover the equilibrium occupation times the total DOS, $G\left(\Sigma_{\alpha }\Gamma_{\alpha }\right)G^{†}=2ℑ\left(G\right)$, which is the total (equilibrium) number of excitations in the device. In the heat current, this term appears as $-\omega C\Gamma_{\alpha }/2$ and can be interpreted as the heat current going from (because of the negative sign) the device into this lead as $\Gamma_{\alpha }/2\omega$ is the escape rate into the lead α.

Similar to the treatment of $Z_{\alpha }$, we will start with the equation of motion, Equation (18), and the FD theorem, Equation (19), to write *C* as:

(27) $$\begin{array}{cc} & C\left(\omega \right)=\langle Y\left(\omega \right)Y^{†}\left(\omega \right)\rangle =\\ & G\left(\omega \right)\left[\Sigma_{\alpha }\left(\langle \eta_{\alpha }\eta_{\alpha }^{†}\rangle +\langle A\eta_{\alpha }^{†}\rangle +\langle \eta_{\alpha }A^{†}\rangle \right)+\langle AA^{†}\rangle \right]G^{†}\left(\omega \right) \end{array}$$

The first term is the NEMF contribution and is reduced to a known result, with now the renormalized Green’s function G being used instead of the standard harmonic one (*G* or G), in order to include thermal effects to some extent:

(28) $$C^{NEMF}\left(\omega \right)=G\;\Sigma_{\alpha }\langle \eta_{\alpha }\eta_{\alpha }^{†}\rangle \;G^{†}=\Sigma_{\alpha }\;\left(G\Gamma_{\alpha }G^{†}\right)\;f_{\alpha }\tau$$

The NEMF approximation, which consists in using G and neglecting the contributions of anharmonicity included in A, is very similar to the harmonic approximation. Within this approximation, where $C\approx C^{NEMF}$ and $Z_{\alpha }\approx Z_{\alpha }^{NEMF}$, the transmission becomes $\mathrm{Tr}\;[G\Gamma_{L}G^{†}\Gamma_{R}]$, very similar to the harmonic result (see Appendix E), but with the GF substituted by the renormalized G, and includes the effect of (quartic) anharmonicity to the lowest order.

Linear terms in A lead to terms similar to $Z_{\alpha }$, which has already been discussed. The only remaining difficulty is with the $\langle AA^{†}\rangle$ terms, which do not explicitly contain any noise term, but have higher powers of displacements. Such terms can only be calculated approximately as the use of the equations of motion will involve higher powers of displacements. To have a second-order approximation consistent with that used for $Z_{\alpha }$ in Equation (26), we have to use:

$${\langle AA^{†}\rangle }^{\left(3\right)}=C^{\left(3\right)}=\frac{1}{4}{\bar{\Psi }}^{2}\sum_{\omega_{1}}C\left(\omega -\omega_{1}\right)C\left(\omega_{1}\right)$$

It can be shown that this approximation, taken to a self-consistent level satisfies current conservation, meaning $\sum_{\alpha }j_{\alpha }=0$, however the spectral components of the current: $ℑZ_{\alpha }\left(\omega \right)-C\left(\omega \right)\Gamma_{\alpha }\left(\omega \right)/2$ do not necessarily lead to zero when summed over all leads. Including for completeness both the cubic and quartic components of the anharmonic forces to second order, the self-consistent set of equations to be solved with the reference Green’s function G are:

(29) $$\begin{array}{cc} & Z_{\alpha }=Z_{\alpha }^{NEMF}+G\left(\Sigma^{\left(3\right)}+\Sigma^{\left(4\right)}\right)Z_{\alpha }\\ & \Sigma^{\left(3\right)}\left(\omega \right)=2{\bar{\Psi }}^{2}\sum_{\omega_{1}}G\left(\omega -\omega_{1}\right)C\left(\omega_{1}\right)\\ & \Sigma^{\left(4\right)}\left(\omega \right)=\frac{3}{2}{\bar{\chi }}^{2}\sum_{\omega_{1},\omega_{2}}G\left(\omega -\omega_{1}-\omega_{2}\right)C\left(\omega_{1}\right)C\left(\omega_{2}\right)\\ & C=\sum_{\alpha }\left(\delta Z_{\alpha }\;G^{†}+G\;\delta Z_{\alpha }^{†}\right)+C^{NEMF}+GPG^{†}\\ & \delta Z_{\alpha }=Z_{\alpha }-Z_{\alpha }^{NEMF}\\ & P=C^{\left(3\right)}+C^{\left(4\right)}\\ & C^{\left(3\right)}=\frac{1}{2}{\bar{\Psi }}^{2}\sum_{\omega_{1}}C\left(\omega -\omega_{1}\right)C\left(\omega_{1}\right)\\ & C^{\left(4\right)}=\frac{1}{6}{\bar{\chi }}^{2}\sum_{\omega_{1},\omega_{2}}C\left(\omega -\omega_{1}-\omega_{2}\right)C\left(\omega_{1}\right)C\left(\omega_{2}\right) \end{array}$$

**Figure A1:**
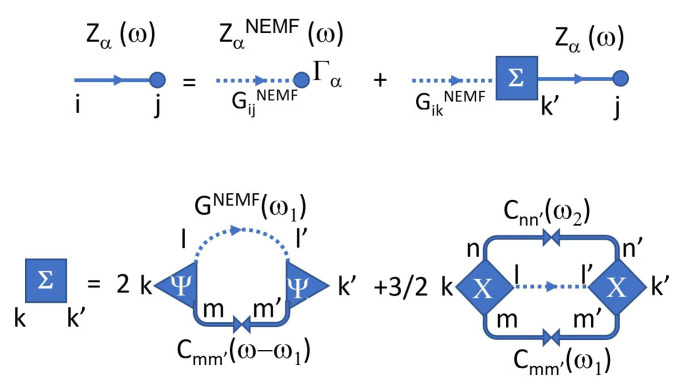
Feynman diagrams associated with $Z_{\alpha }$ up to second order in anharmonic forces. Dashed lines represent the phonon Green’s function G, the circle represents $\Gamma_{\alpha }$, the triangle represents the third-order vertex $\bar{\Psi }$ and the square the fourth-order vertex χ. The thick line with opposite arrows represents the displacement autocorrelation C(ω). Frequency must be conserved at each vertex.

**Figure A2:**
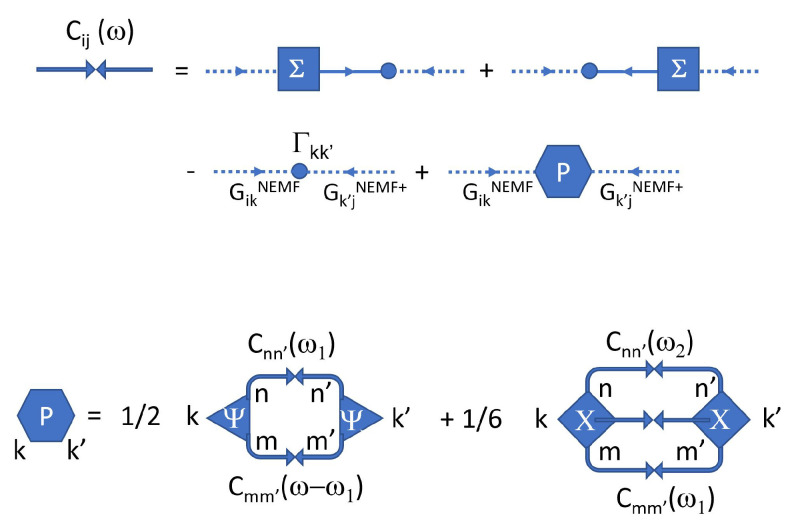
Feynman diagrams associated with C(ω) up to the second order in anharmonic forces. Conventions are the same as in Figure A1.

The equations for $Z_{\alpha }$ and *C* can also be represented using Feynman diagrams as shown in Figure A1 and Figure A2 in Appendix C.3 and Appendix C.4. More explicit forms of these equations are also reproduced in this appendix as Equations (A17) and (A18).

Starting inputs for *C* and $Z_{\alpha }$ could be their NEMF values in the right-hand sides of the above equations, and the latter can be solved iteratively until convergent. Note that while the term $Z_{\alpha }$ requires ∂A/∂Y, the terms *C* require A itself, but these equations contain only second powers of A and ∂A/∂Y. Once iterations converge, the obtained *C* and $Z_{\alpha }$ functions can then be inserted in Equation (13) to compute the heat currents from each lead.

These equations would be the same as the ones obtained from the many-body non-equilibrium Keldysh formalism with the difference that the “occupation factors” $f_{\alpha }=k_{B}T/\omega$ are classical ones, instead of Bose–Einstein functions. In this sense, they can directly be compared to results from classical non-equilibrium MD simulations, which are exact in anharmonicity but have inherent statistical noise in them.

Another interesting feature to note is the increase in the overall conductance with the temperature (if ΔT is held small). This is in agreement with previous MD simulations [13,14].

## 9. Conclusions

To summarize, we developed a self-consistent approximation for transport in anharmonic systems out of equilibrium in the high-temperature (classical) regime. There is therefore no factors of *ℏ* in the formalism and ω is to be interpreted as frequency only, not energy. Although the set of derived equations for the current Equation (13) the equation of motion Equation (18), and the Equations (24) and (27) defining the correlation functions, were formally exact, one has to develop approximations to solve the Dyson’s Equation (24) and the Equation (27) defining *C*. One, because the anharmonic force A is an infinite Taylor expansion and is usually truncated, and two, because its derivative appears in the denominator of Equation (24), which cannot be exactly inverted. In this work, we truncated the Taylor expansion of A up to quartic terms and only included up to second powers of A and ∂A/∂Y in Equations (24) and (27).

We showed that thermal expansion needs to be included using both cubic and quartic terms (to avoid any divergence) and it has the effect of renormalizing FCs as *T* is increased. The reference GF to work with, G, has two corrections: one due to thermal expansion implying changes in bond length and strength ($\bar{\Phi }$ instead of Φ), and the other due to thermal fluctuations about the average position (〈∂A/∂Y〉, which usually involves the quartic term and the autocorrelation *C*), similar in spirit to the self-consistent phonon theory, except that one is not at thermal equilibrium. This is the leading-order anharmonic correction, and cubic anharmonicity contributes to second-order correction terms as shown in the self-consistent equations (29).

Non-equilibrium averages were possible to calculate with the use of the fluctuation–dissipation theorem (Equation (19)), the equations of motion (Equation (18)) and the NFD theorem (Equation (22)).

An alternative approach to investigate non-equilibrium effects at and near interfaces would be to perform a non-equilibrium molecular dynamics simulation (NEMD) of the system attached to thermostats at different temperatures and sample the atomic trajectories in the phase space to find the distribution functions and the position averages—this, however, has inherent noise in it.

One way to extract the effective force constants is to fit from the knowledge of the forces on atoms and their positions in each MD snapshot, the forces to a linear model $F_{i}^{NEMD}\approx F_{i}^{Harmonic}=-{\bar{\Phi }}_{ij}y_{j}$ in order to extract the effective (non-equilibrium) harmonic force constants $\bar{\Phi }$. The remainder can then be defined as the anharmonic force: $F_{i}^{NEMD}=-{\bar{\Phi }}_{ij}y_{j}+a_{i}\left(y\right)$, and the present results may be used. Despite the approximations used in this work, the advantage of this formalism over MD simulations, which includes anharmonicity to all orders, is that it is analytical and therefore fast and free of simulation noise, although reaching self-consistency can be challenging for some model systems. It would be desirable to make a comparison of the results with NEMD to validate these approximations for a given system. The accuracy also relies on the force field and strength of higher-order terms: sources of divergence would be in the denominator of Equation (24) if G〈∂A/∂Y〉≈1, signaling resonances, in which case the Taylor expansion in Equation (24) is not appropriate.

Applications to nanoscale systems will appear in future publications.

## Abbreviations

The following abbreviations are used in this manuscript:

D	Device
DC	Direct Current
DFT	Density Functional Theory
FC	Force Constant
GF	Green’s Function
MD	Molecular Dynamics
NEMD	Non-Equilibrium Molecular Dynamics
NEMF	Non-Equilibrium Mean-Field

## Appendix A. Calculation of Time Averages

When calculating time average of a product such as a(t)b(t) in terms of their Fourier transform, some care needs to be taken:

$$\langle \langle a\left(t\right)b\left(t\right)\rangle \rangle =\frac{1}{\tau }\int_{-\tau /2}^{\tau /2}\langle a\left(t\right)b\left(t\right)\rangle \;dt;\left(\tau \to \infty \right)$$

where one set of brackets is for time averaging and the second set is a thermodynamic average over different initial conditions. To simplify the notations, we have however used only one set of brackets.

In terms of their Fourier transform, we have

$$\langle \langle a\left(t\right)b\left(t\right)\rangle \rangle =\int_{-\tau /2}^{\tau /2}\int \frac{d\omega }{2\pi }\frac{d\omega^{\prime }}{2\pi }\;e^{-i(\omega +\omega^{\prime })t}\;\frac{dt}{\tau }\;\langle A\left(\omega \right)B\left(\omega^{\prime }\right)\rangle$$

However,

$$\langle e^{-i\omega t}\rangle =\int_{-\tau /2}^{\tau /2}\;e^{-i\omega t}\;\frac{dt}{\tau }=\frac{\sin\;\omega \tau /2}{\omega \tau /2}\underset{\tau \to \infty }{\to }\delta_{\omega ,0}\left(=1\;\mathrm{or}\;\;0\right)$$

On the other hand, taking τ→∞ in the boundaries of integral of $e^{-i\omega t}$ leads to 2πδ(ω) which should cancel the τ in the denominator in order to give 1. This means, when taking time averages, one simply needs to take the convolution of the Fourier transforms at Ω=0:

(A1) $$\langle a\left(t\right)b\left(t\right)\rangle =\frac{1}{\tau }\int \frac{d\omega }{2\pi }\;\langle A\left(\omega \right)B\left(-\omega \right)\rangle$$

When calculating diagonal terms of autocorrelations, such as in $\langle \zeta \left(\omega \right)\zeta^{†}\left(\omega \right)\rangle =2\gamma k_{B}T\tau$, the result is proportional to the integration time τ. The latter cancels the τ in the denominator coming from time averaging. Loosely speaking, 2πδ(ω)/τ=1or0. As an example, to calculate the average (DC) current one simply needs to take the diagonal terms in frequencies, omitting factors of τ appearing in the correlation functions:

(A2) $$\langle j_{\alpha }\rangle =\lim_{\overset{\tau \to \infty }{\Omega \to 0}}\frac{\langle J_{\alpha }\left(\Omega \right)\rangle }{\tau }=ℜ\int \frac{d\omega }{2\pi }\frac{\left(-i\omega \right)}{\tau }\left[\langle Y\left(\omega \right)\eta_{\alpha }^{†}\left(\omega \right)\rangle +\langle Y\left(\omega \right)Y^{†}\left(\omega \right)\rangle \;\sigma_{\alpha }^{†}\left(\omega \right)\right]$$

## Appendix B. Change of Position Variables Due to Thermal Expansion

As the temperature of a system is raised, there can the thermal expansion due to anharmonicity. The equilibrium position of the atoms is shifted, and this can also cause a change in the force constants as bond lengths have changed. To take these effects into account, we will slightly modify the formalism as follows:

Let us Taylor-expand the interaction potential in the device in powers of rescaled atomic displacements $x=\sqrt{m}\;u$, about the zero temperature equilibrium positions:

(A3) $$V\left(x_{1},\ldots ,x_{n}\right)=V\left(0,\ldots 0\right)+\frac{1}{2!}\Phi_{ij}\;x_{i}\;x_{j}+\frac{1}{3!}\;\Psi_{ijk}\;x_{i}x_{j}x_{k}+\frac{1}{4!}\;\chi_{ijkl}\;x_{i}x_{j}x_{k}x_{l}+\ldots$$

where, for brevity, we omitted the summation sign over repeated indices, and where the residual force on atom *i* is zero because one starts from a fully relaxed configuration. The coefficients of this expansion can be obtained from a zero-temperature DFT calculation for instance [20,21].

One way, and actually the correct way to compute the non-equilibrium average of the force constants, is to perform a non-equilibrium molecular dynamics (NEMD) simulation in which the system is subject to two or more thermostats at different temperatures and zero pressure. Average positions 〈x〉 maybe computed from the runs, and then one may fit the forces with an effective harmonic model as we will describe below. In a thermally expanded system where 〈x〉 is non-zero, we Taylor expand the potential about the “non-equilibrium” values 〈x〉 instead of zero. This leads to a change in the force constants. The new dynamical variables are chosen so that their average is zero, i.e., they oscillate about the new thermal non-equilibrium positions:

y(t)=x(t)−〈x〉=>Y(ω)=X(ω)−2πδ(ω)〈x〉;〈y〉=0

The average position is defined to be the solution of the average force being zero. In terms of these new variables, and up to the second order in powers of displacements, the force on atom *i* can be written as:

(A4) $$-\frac{\partial V}{\partial x_{i}}=F_{i}=F_{i}\left(x=\langle x\rangle \right)+{\left(\frac{\partial F_{i}}{\partial y_{j}}\right)}_{x=\langle x\rangle }\;y_{j}+\frac{1}{2}{\left(\frac{\partial^{2}F_{i}}{\partial y_{j}y_{k}}\right)}_{x=\langle x\rangle }\;y_{j}y_{k}+\ldots$$

The first term, the residual force, will be used to define the thermal expansion 〈x〉 (by setting the residual or average force to zero: $F_{i}\left(x=\langle x\rangle \right)=-{\left(\frac{\partial V}{\partial y_{i}}\right)}_{x=\langle x\rangle }=0$), which is the Equation (14) in the main text. The second term is the effective, or temperature-dependent, harmonic force, and the last term is the effective anharmonic force. In the case where the potential energy is Taylor-expanded up to quartic order such as in Equation (A3), one can explicitly work out the equation satisfied by 〈x〉, and derive explicit expressions for the effective harmonic and anharmonic FCs. The expression for the force in this case is as follows:

$$\begin{array}{cc} -\frac{\partial V}{\partial x_{i}} & =F_{i}=-\Phi_{ij}x_{j}-\frac{1}{2}\Psi_{ijk}x_{j}x_{k}-\frac{1}{6}\chi_{ijkl}\;x_{j}x_{k}x_{l}-\Sigma_{\alpha }V_{\alpha }\;x_{\alpha }\\ & =-\Phi_{ij}\langle x_{j}\rangle -\frac{1}{2}\Psi_{ijk}\langle x_{j}\rangle \langle x_{k}\rangle -\frac{1}{6}\chi_{ijkl}\langle x_{j}\rangle \langle x_{k}\rangle \langle x_{l}\rangle -\Phi_{ij}y_{j}\\ & -\frac{1}{2}\Psi_{ijk}\left(y_{j}y_{k}+y_{j}\langle x_{k}\rangle +\langle x_{j}\rangle y_{k}\right)\\ & -\frac{1}{6}\chi_{ijkl}\;\left(y_{j}y_{k}y_{l}+3y_{j}y_{k}\;\langle x_{l}\rangle +3y_{j}\langle x_{k}\rangle \langle x_{l}\rangle \right)-\Sigma_{\alpha }V_{\alpha }\;x_{\alpha } \end{array}$$

where use was made of the symmetry of Ψ and χ under permutation of their j,k,l indices.

### Appendix B.1. Thermal Expansion

To find the average positions 〈x〉, we set the average force on each atom *i*, $\langle F_{i}\rangle =-\langle {\left(\partial V/\partial x_{i}\right)}_{x=\langle x\rangle }\rangle$ to zero, and $\langle x_{\alpha }\rangle =0$ (leads are harmonic and will not thermal-expand), and solve for 〈x〉. Thus, the coupling term with the leads, which is linear in lead degrees of freedom and has zero average, will vanish, and we have the following set of non-linear equations in 〈x〉 in terms for force constants and averages of 〈yy〉 and 〈yyy〉:

(A5) $$\begin{array}{ccc} {}[\Phi_{ij}\; & + & \frac{1}{2}\chi_{ijkl}\;\langle y_{k}y_{l}\rangle ]\langle x_{j}\rangle +\frac{1}{2}\Psi_{ijk}\;\langle x_{j}\rangle \langle x_{k}\rangle +\frac{1}{6}\chi_{ijkl}\;\langle x_{j}\rangle \langle x_{k}\rangle \langle x_{l}\rangle \\ & = & -\frac{1}{2}\Psi_{ijk}\;\langle y_{j}y_{k}\rangle -\frac{1}{6}\chi_{ijkl}\;\langle y_{j}y_{k}y_{l}\rangle \end{array}$$

### Appendix B.2. The New Force Constants

The new force constants are simply defined to the be potential derivatives evaluated at the new average positions. Up to quartic order, they are defined as:

$$\begin{array}{c} \begin{array}{cc} {\bar{\Phi }}_{ij} & ={\left(\frac{\partial^{2}V}{\partial x_{i}\partial x_{j}}\right)}_{\langle x\rangle }=\Phi_{ij}+\Psi_{ijk}\;\langle x_{k}\rangle +\frac{1}{2}\chi_{ijkl}\;\langle x_{k}\rangle \langle x_{l}\rangle \\ {\bar{\Psi }}_{ijk} & ={\left(\frac{\partial^{3}V}{\partial x_{i}\partial x_{j}\partial x_{k}}\right)}_{\langle x\rangle }=\Psi_{ijk}+\chi_{ijkl}\;\langle x_{l}\rangle \\ {\bar{\chi }}_{ijkl} & =\chi_{ijkl} \end{array} \end{array}$$

Finally, the average positions can be re-expressed in terms of the new force constants as:

(A6) $$\begin{array}{c} {\bar{\Phi }}_{ij}\;\langle x_{j}\rangle -\frac{1}{2}{\bar{\Psi }}_{ijk}\;\langle x_{j}\rangle \langle x_{k}\rangle +\frac{1}{6}{\bar{\chi }}_{ijkl}\;\langle x_{j}\rangle \langle x_{k}\rangle \langle x_{l}\rangle =-\frac{1}{2}{\bar{\Psi }}_{ijk}\;\langle y_{j}y_{k}\rangle -\frac{1}{6}{\bar{\chi }}_{ijkl}\;\langle y_{j}y_{k}y_{l}\rangle \end{array}$$

These coupled set of non-linear equations (for all *i*) in ($\langle x\rangle ,\bar{\Phi },\bar{\Psi },\bar{\chi }$ must be solved in terms of 〈yy〉 and 〈yyy〉 covariance matrices, to find the average positions and effective force constants. It requires a self-consistent iterative solution as the 〈yy〉 correlations will in turn depend on 〈x〉.

### Appendix B.3. New Equations of Motion

Finally, changing to new dynamical variables y(t) and new force constants, the equation of motion for *y* becomes:

(A7) $$\begin{array}{cc} \frac{d^{2}y_{i}}{dt^{2}} & =-{\left(\frac{\partial^{2}V}{\partial y_{i}\partial y_{j}}\right)}_{y=0}\;y_{j}-\frac{1}{2}{\left(\frac{\partial^{3}V}{\partial y_{i}\partial y_{j}\partial y_{k}}\right)}_{y=0}\;y_{j}y_{k}\\ & -\frac{1}{6}{\left(\frac{\partial^{4}V}{\partial y_{i}\partial y_{j}\partial y_{k}\partial y_{l}}\right)}_{y=0}\;y_{j}y_{k}y_{l}-\ldots -\Sigma_{\alpha }V_{\alpha }\;x_{\alpha }\\ & =-{\bar{\Phi }}_{ij}\;y_{j}-\frac{1}{2}{\bar{\Psi }}_{ijk}\;y_{j}y_{k}-\frac{1}{6}{\bar{\chi }}_{ijkl}\;y_{j}y_{k}y_{l}-\Sigma_{\alpha }V_{\alpha }\;x_{\alpha } \end{array}$$

Adopting this change of variable, the GF keeps the same form with Φ substituted by $\bar{\Phi }$, and anharmonic forces in the right-hand side of the equation of motion become by definition:

(A8) $$a\overset{\mathrm{def}}{=}-\frac{1}{2}{\bar{\Psi }}_{ijk}\;y_{j}y_{k}-\frac{1}{6}{\bar{\chi }}_{ijkl}\;y_{j}y_{k}y_{l}$$

## Appendix C. Explicit Form of the Correlation Functions including the Atomic and Cartesian Indices

In this section, we will give explicit formulas for the correlation functions needed in the average heat current expression in Equation (A2). First let us label by roman letters i,j,k the dynamical degrees of freedom in the device. If there are *N* atoms in the device in three dimensions, each of these labels refers to an atom and one of the three Cartesian components of its displacements. It therefore varies from 1 to 3*N*. As a result the matrices *C*, $\sigma_{\alpha }$ and $Z_{\alpha }$ are 3N×3N matrices. Taking their trace in Formula (A2) gives the heat current, which is a scalar. Note that since transport is along the length of the leads, which are one-dimensional, in principle the components of the power perpendicular to the lead direction should yield zero. More specifically, if the current in lead α is given by $J_{\alpha }=\langle \dot{x}F_{x}+\dot{y}F_{y}+\dot{z}F_{z}\rangle$, and the lead is infinite along the *z* direction for instance, then we should have $J_{\alpha }=\langle \dot{z}F_{z}\rangle$ and $\langle \dot{x}F_{x}\rangle =\langle \dot{y}F_{y}\rangle =0$. So in each lead, one may take three partial traces along and perpendicular to the lead direction and confirm these relations. The total trace should still yield the correct result. One final note is the matrices of ${\bar{\Phi }}_{ij}$ and ${\bar{\Psi }}_{ijk}$ are invariant under permutations of their indices.

### Appendix C.1. Lead Self-Energies σ α and Escape Rates Γ α

The surface Green functions of the leads were defined by an inverse, as stated in Equation (9). The self-energy for lead α is accordingly defined as $\sigma_{\alpha ,ij}\left(\omega \right)=V_{\alpha ,ik}\;\gamma_{\alpha ,kk^{\prime }}\left(\omega \right)\;V_{\alpha ,jk^{\prime }}$ where the two indices $\left(k,k^{\prime }\right)$ refer to the lead degrees of freedom. Note the matrices $V_{\alpha }$ connecting the device to lead α need not be square.

The escape rates were defined by:

(A9) $$\Gamma_{\alpha ,ij}=-i\left(\sigma_{\alpha ,ij}-\sigma_{\alpha ,ij}^{†}\right)=2ℑ\left(\sigma_{\alpha ,ij}\right)$$

For Langevin thermostats with white noise, these results do not depend on the thermostat damping factors γ.

### Appendix C.2. Noise Autocorrelation Functions

Let us start with the noise autocorrelation that will appear in the calculation of displacement-noise correlation $Z_{\alpha }$. This autocorrelation can be obtained from the fluctuation–dissipation theorem, a relation that has to hold if the noise is such that the leads are to behave as a Langevin thermostat at temperature *T*:

$$\langle \zeta_{\alpha }\left(t\right)\zeta_{\alpha^{\prime }}^{T}\left(t^{\prime }\right)\rangle \overset{FD}{=}2\gamma_{\alpha }k_{B}T_{\alpha }\;\delta \left(t-t^{\prime }\right)\delta_{\alpha ,\alpha^{\prime }}$$

The noise is white and different sites are uncorrelated with each other. This relation implies that in the frequency domain the autocorrelation of ζ and that of η satisfy the following relations:

(A10) $$\langle \zeta_{\alpha }\left(\omega \right)\zeta_{\alpha^{\prime }}^{T}\left(\omega^{\prime }\right)\rangle =2\pi \delta \left(\omega +\omega^{\prime }\right)\;\delta_{\alpha ,\alpha^{\prime }}\;2\gamma_{\alpha }k_{B}T_{\alpha }$$

(A11) $$\begin{array}{cc} & \langle \eta_{\alpha }\left(\omega \right)\;\eta_{\alpha^{\prime }}^{T}\left(\omega^{\prime }\right)\rangle =V_{\alpha }g_{\alpha }\;,\langle \zeta_{\alpha }\zeta_{\alpha^{\prime }}^{T}\rangle \;g_{\alpha^{\prime }}^{T}V_{\alpha^{\prime }}^{T} \end{array}$$

(A12) $$=2\pi \delta \left(\omega +\omega^{\prime }\right)\delta_{\alpha ,\alpha^{\prime }}\;k_{B}T_{\alpha }\;\left[V_{\alpha }g_{\alpha }\;\left(2\gamma_{\alpha }\right)\;g_{\alpha }^{T}V_{\alpha }^{T}\right]$$

In the above, $g_{\alpha }$ is a diagonal matrix of size equal to the number of degrees of freedom in the lead α (which is infinity!) At this point, we will use a convenient identity satisfied by any Green’s function *G*. If $G^{-1}=a+ib$, with (a,b) real matrices, then

(A13) $$i\left(G-G^{†}\right)=iG\left(\frac{1}{G^{†}}-\frac{1}{G}\right)G^{†}=G\;\left(2b\right)\;G^{†}$$

Since $ℑ\left(g_{\alpha }^{-1}\right)=-\omega \gamma_{\alpha }$ we can write the η-autocorrelation as:

(A14) $$\begin{array}{c} \langle \eta_{\alpha }\left(\omega \right)\;\eta_{\alpha^{\prime }}^{T}\left(\omega^{\prime }\right)\rangle =-iV_{\alpha }\left(g_{\alpha }-g_{\alpha }^{†}\right)V_{\alpha }^{T}\times 2\pi \delta \left(\omega +\omega^{\prime }\right)\delta_{\alpha ,\alpha^{\prime }}\;\frac{k_{B}T_{\alpha }}{\omega }\\ =-i\left(\sigma_{\alpha }-\sigma_{\alpha }^{†}\right)\times 2\pi \delta \left(\omega +\omega^{\prime }\right)\delta_{\alpha ,\alpha^{\prime }}\;\frac{k_{B}T_{\alpha }}{\omega }=\Gamma_{\alpha }\times 2\pi \delta \left(\omega +\omega^{\prime }\right)\delta_{\alpha ,\alpha^{\prime }}\;\frac{k_{B}T_{\alpha }}{\omega } \end{array}$$

where we used the notation $\Gamma_{\alpha }=-i\left(\sigma_{\alpha }-\sigma_{\alpha }^{†}\right)=2ℑ\left(\sigma_{\alpha }\right)$ for twice the imaginary part of the lead α self-energy. Alternatively, the diagonal elements in frequency can be written as:

(A15) $$\begin{array}{c} \langle \eta_{\alpha }\left(\omega \right)\eta_{\alpha^{\prime }}^{T}\left(-\omega \right)\rangle =\Gamma_{\alpha }\times \delta_{\alpha ,\alpha^{\prime }}\;\frac{k_{B}T_{\alpha }}{\omega }\tau \end{array}$$

where τ is the integration time which goes to infinity. Dhar and Roy [22] have shown that in the quantum limit, when taking a semi-infinite harmonic lead and averaging over all possible initial conditions sampled from a canonical ensemble, the quantum noise term has an autocorrelation given by

$$\langle \eta_{\alpha }\left(\omega \right)\eta_{\alpha^{\prime }}^{T}\left(\omega^{\prime }\right)\rangle =h\Gamma_{\alpha }\delta \left(\omega +\omega^{\prime }\right)\delta_{\alpha ,\alpha^{\prime }}\left(1+f(\hbar \omega /k_{B}T)\right)$$

where $f\left(x\right)={\left[e^{x}-1\right]}^{-1}$ is the equilibrium Bose–Einstein distribution function, which, in the classical (high-temperature) limit, reduces to $k_{B}T/\hbar \omega$. Our classical result based on properties of Langevin thermostats is thus consistent with the quantum one.

We can note that the explicit dependence on the damping factor $\gamma_{\alpha }$ has been replaced by twice the imaginary part of the lead self-energy $\Gamma_{\alpha }$, which one may interpret as 2ω times a “rate”. For the adopted white noise, the dependence on its damping factor has gone away! Furthermore, in the calculation of $Z_{\alpha }$, the notation $\langle \eta \eta^{†}\rangle$ implies the diagonal terms $\omega^{\prime }=-\omega$ must be taken. Using the Novikov–Furutsu–Donsker (NFD) identity (see Appendix D) we can see that $\langle A\eta^{†}\rangle \propto \langle \eta \eta^{†}\rangle$ and therefore only diagonal terms in frequency will appear in the frequency integral of the heat current, and assuming thermostat temperatures are steady, the heat current can only have a DC (Ω=0) component. Note that this component is infinite the way we defined it: $J_{\alpha }\left(\Omega =0\right)=\int_{-\infty }^{\infty }\langle j_{\alpha }\left(t\right)\rangle \;dt$. To find the average heat current, we have to divide this by the integration time τ, and then take the limit τ→∞. This division cancels the τ appearing in the numerator of noise autocorrelations. This issue is further discussed in the Appendix A, Equation (A2). Final results do not depend on τ.

The factor $2\pi \delta (\omega +\omega^{\prime })/\tau$ can now be excluded from the current as it is essentially 1 for the diagonal terms, and zero otherwise.

(A16) $$\begin{array}{c} \langle \eta_{\alpha }\left(\omega \right)\eta_{\alpha }^{†}\left(\omega \right)\rangle =\Gamma_{\alpha }\left(\omega \right)\frac{k_{B}T_{\alpha }}{\omega }\tau =2ℑ\left(\sigma_{\alpha }\left(\omega \right)\right)\frac{k_{B}T_{\alpha }}{\omega }\tau \end{array}$$

### Appendix C.3. Noise-Displacement Correlations Z α

Using the equation of motion (18), the expression defining this correlation function to second order in ∂A/∂Y can be derived to satisfy Equation (25). After substitution, the explicit relations become:

(A17) $$\begin{array}{cc} & Z_{\alpha ,ij}\left(\omega \right)=Z_{\alpha ,ij}^{NEMF}\left(\omega \right)+G_{ik}\left(\omega \right)\;\left(\Sigma_{kk^{\prime }}^{\left(3\right)}+\Sigma_{kk^{\prime }}^{\left(4\right)}\right)\;Z_{\alpha ,k^{\prime }j}\left(\omega \right)\\ & Z_{\alpha ,ij}^{NEMF}\left(\omega \right)=G_{ik}\left(\omega \right)\;\Gamma_{\alpha ,kj}\left(\omega \right)\frac{k_{B}T_{\alpha }}{\omega }\\ & \Sigma_{kk^{\prime }}^{\left(3\right)}\left(\omega \right)=2{\bar{\Psi }}_{klm}\;{\bar{\Psi }}_{k^{\prime }l^{\prime }m^{\prime }}\left(\int_{1}G_{ll^{\prime }}\left(\omega -\omega_{1}\right)\;C_{mm^{\prime }}\left(\omega_{1}\right)\right)\\ & \Sigma_{kk^{\prime }}^{\left(4\right)}\left(\omega \right)=\frac{3}{2}{\bar{\chi }}_{klmn}\;{\bar{\chi }}_{k^{\prime }l^{\prime }m^{\prime }n^{\prime }}\left(\int_{1,2}G_{ll^{\prime }}\left(\omega -\omega_{1}-\omega_{2}\right)\;C_{mm^{\prime }}\left(\omega_{1}\right)\;C_{nn^{\prime }}\left(\omega_{2}\right)\right) \end{array}$$

where, as usual, the summation over repeated indices is implied.

### Appendix C.4. Displacement Autocorrelations C(ω)

These functions, whose trace represents the number of excitations in the device, are defined via Equation (27). The static distortion in 〈x〉〈x〉 does not contribute to the current and will thus be omitted. To the second order in A, the expression for *C* is given by:

(A18) $$\begin{array}{cc} C_{ij}\left(\omega \right)= & C_{ij}^{NEMF}\left(\omega \right)+\sum_{\alpha }\left(Z_{\alpha ,ik}-Z_{\alpha ,ik}^{NEMF}\right)G_{kj}^{†}+G_{ik}{\left(Z_{\alpha ,kj}-Z_{\alpha ,kj}^{NEMF}\right)}^{†}\\ & +G_{ik}\;\left(C_{kk^{\prime }}^{\left(3\right)}+C_{kk^{\prime }}^{\left(4\right)}\right)\;G_{k^{\prime }j}^{†}\\ C_{ij}^{NEMF}\left(\omega \right) & =\Sigma_{\alpha }\;\left(G_{ik}\;\Gamma_{\alpha ,kk^{\prime }}\;G_{k^{\prime }j}^{†}\right)\;\frac{k_{B}T_{\alpha }}{\omega }\\ C_{kk^{\prime }}^{\left(3\right)}\left(\omega \right)= & \frac{1}{2}{\bar{\Psi }}_{klm}\;{\bar{\Psi }}_{k^{\prime }l^{\prime }m^{\prime }}\left(\int_{1}C_{ll^{\prime }}\left(\omega -\omega_{1}\right)\;C_{mm^{\prime }}\left(\omega_{1}\right)\right)\\ C_{kk^{\prime }}^{\left(4\right)}\left(\omega \right)= & \frac{1}{6}{\bar{\chi }}_{klmn}\;{\bar{\chi }}_{k^{\prime }l^{\prime }m^{\prime }n^{\prime }}\left(\int_{1,2}C_{ll^{\prime }}\left(\omega -\omega_{1}-\omega_{2}\right)\;C_{mm^{\prime }}\left(\omega_{1}\right)\;C_{nn^{\prime }}\left(\omega_{2}\right)\right) \end{array}$$

## Appendix D. Statement and Proof of the Novikov–Furutsu–Donsker (NFD) Relation

The Novikov–Furutsu–Donsker relation [19,23,24,25] relates the correlation function of any functional A[η] of the noise η to the noise–noise correlation times the expectation value of the derivative of that functional:

(A19) $$\langle A\left[\eta \right]\;\eta_{\alpha }^{†}\left(t\right)\rangle =\sum_{\beta }\int dt^{\prime }\langle \eta_{\beta }\left(t^{\prime }\right)\eta_{\alpha }^{†}\left(t\right)\rangle \langle \frac{\delta A[\eta ]}{\delta \eta_{\beta }\left(t^{\prime }\right)}\rangle$$

implying that in the frequency domain, we have

(A20) $$\langle A_{i}\left[\eta \right]\;\eta_{j\alpha }^{†}\left(\omega \right)\rangle =\sum_{l,\beta }\langle \frac{\delta A_{i}\left[\eta \right]}{\delta \eta_{l,\beta }}\left(\omega \right)\rangle \langle \eta_{l,\beta }\left(\omega \right)\eta_{j,\alpha }^{†}\left(\omega \right)\rangle$$

**Proof** **of** **the** **NFD** **relation:** Let η be a random variable with Gaussian distribution of mean 0 and variance $\sigma^{2}$. Consider the average S=〈A[η]η〉 where *A* is a functional of η. By definition, $S=\int_{-\infty }^{+\infty }d\eta \frac{e^{-\eta^{2}/2\sigma^{2}}}{\sqrt{2\pi \sigma^{2}}}\eta A\left(\eta \right)$. After performing an integration by parts, we find:

(A21) $$\begin{array}{ccc} S & = & \int_{-\infty }^{+\infty }-\sigma^{2}\frac{1}{\sqrt{2\pi \sigma^{2}}}d\left(e^{-\eta^{2}/2\sigma^{2}}\right)A\left(\eta \right)=\sigma^{2}\int_{-\infty }^{+\infty }\frac{1}{\sqrt{2\pi \sigma^{2}}}e^{-\eta^{2}/2\sigma^{2}}\frac{dA(\eta )}{d\eta }d\eta \\ & = & \sigma^{2}\langle \frac{dA(\eta )}{d\eta }\rangle =\langle \eta \eta \rangle \langle \frac{dA(\eta )}{d\eta }\rangle \end{array}$$

This can easily be extended to higher dimensions where η is an array:

$$\langle A\left[\eta \right]\eta_{i}\rangle =\sum_{j}\langle \frac{\partial A}{\partial \eta_{j}}\rangle \langle \eta_{j}\eta_{i}\rangle$$

□

## Appendix E. Heat Current within the Harmonic Approximation

For a harmonic system (G=G=G), we have derived the following expressions:

(A22) $$\begin{array}{cc} Z_{\alpha }^{H}\left(\omega \right) & =\langle Y\eta_{\alpha }^{†}\rangle =G\Gamma_{\alpha }\frac{k_{B}T_{\alpha }}{\omega }\tau ; \end{array}$$

(A23) $$C^{H}\left(\omega \right)=\langle YY^{†}\rangle =\Sigma_{\beta }\left(G\Gamma_{\beta }G^{†}\right)\frac{k_{B}T_{\beta }}{\omega }\tau$$

The frequency in the denominator will cancel the frequency in the current coming from the time derivative of positions, so that the harmonic current becomes:

$$\langle j_{\alpha }^{H}\rangle =k_{B}\int \frac{d\omega }{2\pi }\;\mathrm{Tr}\;\left(ℑ\left(G\right)\;\Gamma_{\alpha }T_{\alpha }-\Sigma_{\beta }\left(G\;\Gamma_{\beta }T_{\beta }\;G^{†}\right)\frac{\Gamma_{\alpha }}{2}\;\right)$$

Below we will show that this current satisfies both detailed balance and current conservation.

Detailed balance: If all leads are at the same temperature, using Equation (A13), we have

(A24) $$\mathrm{Tr}\;\Gamma_{\alpha }\;ℑ\left(G\right)=\frac{1}{2}\mathrm{Tr}\;\Gamma_{\alpha }\Sigma_{\beta }\left(G\Gamma_{\beta }G^{†}\right)$$

Making this substitution for ℑ(G) in the heat current formula, we end up with:

(A25) $$\langle j_{\alpha }^{H}\rangle =\frac{k_{B}}{2}\int \frac{d\omega }{2\pi }\;\mathrm{Tr}\;\Gamma_{\alpha }\;G\;\left(\Sigma_{\beta }\Gamma_{\beta }\left(T_{\alpha }-T_{\beta }\right)\right)G^{†}$$

This is equation manifestly shows detailed balance as it is linear in temperature differences. It is also easy to see that this harmonic part of the heat current satisfies current conservation, due to the antisymmetric (under exchange of α and β) form of the following sum:

(A26) $$\Sigma_{\alpha }\langle j_{\alpha }^{H}\rangle =\frac{k_{B}}{2}\int \frac{d\omega }{2\pi }\;\Sigma_{\alpha \beta }\;\left(T_{\alpha }-T_{\beta }\right)\;\mathrm{Tr}\;\left[\Gamma_{\alpha }\;G\;\Gamma_{\beta }G^{†}\right]=0$$

In a two-terminal device geometry (β=R;α=L), the expression for the current reduces to the well-known formula:

(A27) $$\langle j_{L}^{H}\rangle =\frac{k_{B}}{2}\left(T_{L}-T_{R}\right)\int \frac{d\omega }{2\pi }\;\mathrm{Tr}\;\left[\Gamma_{L}\;G\;\Gamma_{R}\;G^{†}\right]=-\langle j_{R}^{H}\rangle$$

In general, $\mathrm{Tr}\;[\Gamma_{\alpha }\;G\;\Gamma_{\beta }\;G^{†}]$ maybe interpreted as the harmonic transmission from lead α to lead β. We see that even in the non-equilibrium regime (large ΔT), the harmonic approximation leads to the same transmission function regardless of how large the temperature difference is.

A simple extension to quantum case: Given the correspondence between the quantum and classical versions of noise autocorrelation, to recover the quantum limit, one may replace $f=k_{B}T/\omega$ by $\hbar (1+f_{\mathrm{BE}}\left(\hbar \omega /k_{B}T\right))$. The constant term 1 is irrelevant and disappears due to the principle of detailed balance, and the temperature difference is replaced by the difference in the distribution functions times the phonon energy:$\;k_{B}\left(T_{\alpha }-T_{\beta }\right)<=>\hbar \omega \left(f_{\alpha }-f_{\beta }\right)$.
